# Benefit of the Reduced Dose Combination of Azacitidine and Venetoclax in an Elderly Patient With Acute Myeloid Leukemia

**DOI:** 10.7759/cureus.39481

**Published:** 2023-05-25

**Authors:** Keita Fujino, Hiroshi Ureshino, Tetsumi Yoshida, Tatsuo Ichinohe

**Affiliations:** 1 Hematology and Oncology, Research Institute for Radiation Biology and Medicine, Hiroshima University, Hiroshima, JPN

**Keywords:** bcl-2, elderly population, venetoclax, azacitidine, acute myeloid leukemia (aml)

## Abstract

Elderly patients with acute myeloid leukemia (AML) have been found to clinically benefit from the combination of azacitidine (AZA) and venetoclax (VEN), although the safety and efficacy of the treatment in extremely elderly patients (age >85 years) have not been fully established. An 88-year-old woman diagnosed with AML was given a lower dose of AZA and VEN. She eventually developed grade 4 hypokalemia, necessitating treatment interruption. However, a lower dose of VEN was successfully continued in the subsequent cycle of treatment, resulting in complete remission. Hence, reduced AZA and VEN doses may be beneficial for extremely elderly AML patients.

## Introduction

Acute myeloid leukemia (AML) is a clonal hemopoietic stem and progenitor cell disorder caused by acquired and/or occasionally inherited genetic and/or epigenetic abnormalities [1]. The median age of AML diagnosis is 68 years. Elderly patients are more likely to be diagnosed with AML with myelodysplasia-related changes (MRC), have poor-risk karyotypes, and generally have multiple comorbidities, resulting in lower rates of complete remission, relapse-free survival, and overall survival when compared to younger patients with AML [2,3]. Hypomethylating agents (or HMAs, such as azacitidine, or AZA, and decitabine) or low-dose cytarabine (LDAC) with additional venetoclax (VEN), a selective B-cell lymphoma 2 inhibitor, reportedly yielded clinical benefit for those AML patients, so VEN plus HMAs or LDAC became a standard treatment strategy [4-6]. Although the treatment is well tolerated in the elderly, treatment interruption of VEN or dose reduction of HMAs or LDAC is frequently required due to adverse events, for example, myelosuppression [7]. However, proper management of the combination treatment is not fully understood, particularly in extremely elderly patients (age >85 years) [8]. Here, we present the case of an extremely elderly patient with AML-MRC who was successfully treated with a reduced dose of AZA and VEN.

## Case presentation

An 88-year-old woman came to our hospital for a routine medical checkup after being diagnosed with myelodysplastic syndrome (5q- syndrome) in November 2022. She had previously received lenalidomide and maintained hematological improvement (transfusion independent) for approximately three years (from 2018 to 2020). Thereafter, lenalidomide was discontinued due to the persistence of neutropenia in March 2020, and she was monitored. The gradual development of anemia necessitating red blood cell transfusion and myeloblasts in peripheral blood was observed in July 2022. Lenalidomide was resumed, but it was ineffective at that time, so myeloblast was gradually increased. A complete blood count revealed a decrease in the white blood cell count with an increase in myeloblasts (1.80 × 10^9^/L, with 28% myeloblasts), anemia (hemoglobin, 65 g/L), and mild thrombocytopenia (platelet count, 101 × 10^9^/L). Laboratory tests revealed that the creatinine level was only slightly elevated (0.82 mg/dL). A bone marrow aspirate revealed mild hypercellularity, with 27.6% myeloblasts and multilineage dysplasia. A complex karyotype was revealed by G-banded metaphase analysis, which included del 5q- (47, XX, +1, der(1:7)(q10;p10), del(5)(q?), +8 [16/20], 46, XX [4/20]). Hence, the patient was diagnosed with AML-MRC. We decided to administer AZA and VEN for the treatment of AML-MRC because she was very old, but did not have obvious organ damage or cognitive dysfunction.

Given her age, she received a lower dose of AZA (50 mg/body for five days) with a slow ramp-up and dose reduction of VEN (100 mg on days 1-7, 200 mg on days 7-28). Micafungin was also administered as anti-fungal prophylaxis. Non-hematological adverse events of grade 3 or higher, tumor lysis syndrome, and febrile neutropenia were not documented. Myeloblasts in the peripheral blood gradually decreased and disappeared after the completion of the initial course of AZA and VEN. On day 28, bone marrow aspiration revealed residual myeloblasts (26.4%).

Subsequently, the second course of AZA and VEN was started. We reduced the dose of VEN (20 mg/day) due to the use of posaconazole (CYP3A4 inhibitor) as anti-fungal prophylaxis (when using a strong CYP3A4 inhibitor, the venetoclax dose of 200 mg without the CYP3A4 inhibitor would be similar to 20 mg). She was discharged following the completion of the second course of AZA (50 mg/day for five days). On day 18, hyperbilirubinemia (grade 1, total bilirubin level 1.6 mg/dL) and hypokalemia (grade 2, potassium 2.6 mEq/L) occurred, so VEN was reduced to 10 mg/day and spironolactone was added to preserve potassium. However, on day 21, she developed grade 4 hypokalemia (2.0 mEq/L). Unusual diets leading to hypokalemia were not identified. She was admitted and VEN was discontinued. Potassium was supplemented via intravenous infusion and oral intake. On day 24, serum bilirubin concentrations returned to normal, and on day 30, serum potassium levels returned to normal.

**Figure 1:**
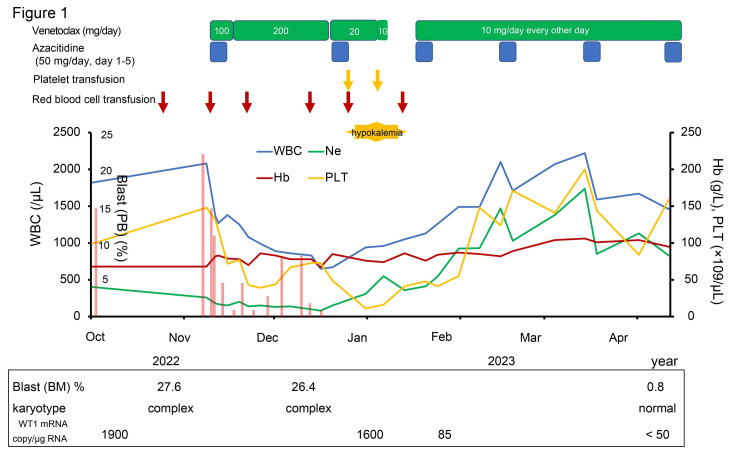
Clinical course of the patient WBC, white blood cell; Hb, hemoglobin; PLT, platelet

The third course of AZA and VEN was resumed on day 33 after the second course of AZA and VEN. VEN was administered at a reduced dose of 10 mg every other day. During the third course of AZA and VEN without potassium supplementation, hyperbilirubinemia and hypokalemia were not observed with gradual blood cell recovery that was transfusion independent (several red blood cell or platelet transfusions were required in the first or second course of the treatment; Figure 1). The fourth and fifth AZA and VEN courses were completed in a 28-day cycle without severe adverse events. Bone marrow aspiration after the fifth course of AZA and VEN revealed that she had achieved complete remission with incomplete count recovery (CRi, myeloblasts 0.8% in bone marrow) and further achieved cytogenetic response (G-banding: 46, XX [4/4]). WT1 mRNA levels in peripheral blood were also normalized (<50 copies/μg RNA). Now, the sixth course of AZA and VEN is currently underway (Figure 1).

## Discussion

The patient was successfully treated with a reduced dose of AZA and VEN for AML-MRC, even though the patient was very old (age >85 years). Intensive chemotherapy generally does not benefit most elderly patients with AML because of their low response or survival rate; therefore, thus less intensive chemotherapy is preferred in these patients [9]. The combination of HMAs or LDAC and VEN has been considered the standard treatment for patients with AML who are transplant ineligible, because of its high efficacy and low toxicity rate, particularly in elderly patients with AML-MRC and adverse cytogenetic abnormalities [4-6]. The treatment is feasible for patients with AML who have severe comorbidities [10]. Nonetheless, the median age of patients in the previous studies was 76 years (the oldest was aged 93 years); as a result, the treatment's safety and efficacy in extremely elderly patients (age >85 years) has not been fully elucidated.

﻿Extremely elderly patients with AML were frequently assigned the best supportive care (BSC) based on their performance status and serious comorbidities. However, the survival outcomes in patients who received only BSC were very poor [11]. Thus, some therapeutic intervention is needed for these patients. ﻿The use of HMAs reportedly may be beneficial for extremely elderly patients compared with BSC [11]. Several assessment methods have evaluated fitness in elderly AML patients for chemotherapy, considering their comorbidities [12]. Our patient had no obvious cardiac, pulmonary, hepatic, or cognitive dysfunction (only had mild renal dysfunction); thus, we performed AZA and VEN therapy for the patient. Indeed, the patient achieved CRi with a cytogenetic response and was discharged for transfusion dependency, implying that AZA and VEN treatment could provide substantial benefits in terms of both survival and quality of life in this patient.

In contrast, the patient developed grade 4 hypokalemia as a result of severe adverse events. Hypokalemia of grade 3 or higher was found to be common in patients receiving LDAC or HMAs and VEN treatment (approximately 10%-20%) [4-6,13]. Aging is associated with a decreased drug metabolism capacity in the liver [14]. When using a CYP3A4 inhibitor, venetoclax dose should be reduced for the prevention of increased serum concentration, while the effects of CYP3A4 inhibition are varied for each individual. CYP3A4 might be strongly inhibited by posaconazol, leading to a high serum concentration of venetoclax despite a dose reduction from 200 to 20 mg. Unfortunately, serum VEN and posaconazole concentrations cannot be assessed in a general clinical setting in Japan. The current patient’s hypokalemia was successfully treated by lowering the VEN dose. Given that VEN metabolism is dependent on hepatic enzymatic activities, physicians should take this into account when modifying administration schedules and dosage in elderly patients, as there is no standard VEN dose reduction strategy [15].

## Conclusions

Here, we have presented the case of an extremely elderly patient with AML-MRC who was successfully treated with a reduced dose of AZA and VEN. An appropriate dose reduction method for AZA and VEN must be established to administer AZA and VEN safely in extremely elderly AML patients with reduced organ function. We believe this report will encourage the hematologists to establish a dose reduction strategy for AZA + VEN.
